# Physiological concentrations of cyanide stimulate mitochondrial Complex IV and enhance cellular bioenergetics

**DOI:** 10.1073/pnas.2026245118

**Published:** 2021-05-10

**Authors:** Elisa B. Randi, Karim Zuhra, Laszlo Pecze, Theodora Panagaki, Csaba Szabo

**Affiliations:** ^a^Chair of Pharmacology, Faculty of Science and Medicine, University of Fribourg, 1700 Fribourg, Switzerland

**Keywords:** mitochondria, bioenergetics, gasotransmitters

## Abstract

In mammalian cells, cyanide is viewed as a cytotoxic agent, which exerts its effects through inhibition of mitochondrial Complex IV (Cytochrome C oxidase [CCOx]). However, the current report demonstrates that cyanide’s effect on CCOx is biphasic; low (nanomolar to low-micromolar) concentrations stimulate CCOx activity, while higher (high-micromolar) concentrations produce the “classic” inhibitory effect. Low concentrations of cyanide stimulated mitochondrial electron transport and elevated intracellular adenosine triphosphate (ATP), resulting in the stimulation of cell proliferation. The stimulatory effect of cyanide on CCOx was associated with the removal of the constitutive, inhibitory glutathionylation on its catalytic 30- and 57-kDa subunits. Transfer of diluted *Pseudomonas aeruginosa* (a cyanide-producing bacterium) supernatants to mammalian cells stimulated cellular bioenergetics, while concentrated supernatants were inhibitory. These effects were absent with supernatants from mutant *Pseudomonas* lacking its cyanide-producing enzyme. These results raise the possibility that cyanide at low, endogenous levels serves regulatory purposes in mammals. Indeed, the expression of six putative mammalian cyanide-producing and/or -metabolizing enzymes was confirmed in HepG2 cells; one of them (myeloperoxidase) showed a biphasic regulation after cyanide exposure. Cyanide shares features with “classical” mammalian gasotransmitters NO, CO, and H_2_S and may be considered the fourth mammalian gasotransmitter.

Mitochondrial respiration is responsible for the majority of adenosine triphosphate (ATP) generation in eukaryotes. Electron transport along the mitochondrial electron transport chain complexes I, II, III, and IV creates an electrochemical proton gradient, which acts as the driving force for ATP generation (1). Cyanide has been long recognized as an inhibitor of mitochondrial electron transport due to its binding to the heme a3 prosthetic group in Complex IV (Cytochrome C oxidase [CCOx]). The shutdown of aerobic ATP generation is recognized as the primary mode of cyanide’s cytotoxic action in eukaryotes (1).

Although cyanide in eukaryotes is predominantly viewed as a toxic molecule, various mammalian enzymes are known to produce cyanide, thereby maintaining nanomolar to low-micromolar physiological cyanide levels (2–5). We have therefore evaluated the potential effect of a broad concentration range of cyanide on mitochondrial function and cell proliferation in human cells. Experiments in cultured HepG2 hepatocytes revealed that cyanide at low (0.1-nM to 1-µM) concentrations stimulates mitochondrial electron transport and oxygen consumption, while higher (10-µM and above) concentrations produce the well-established (1) inhibitory effects (Fig. 1 *A* and *B*). Low and high concentrations of cyanide increased or decreased, respectively, cellular ATP levels, as demonstrated by two independent methods: extracellular flux analysis (Fig. 1*A*) and a ratiometric intracellular ATP:adenosine diphosphate (ADP) fluorescent biosensor (Fig. 1*C*). The activity of Complexes I, II, and III was largely unaffected by cyanide (Fig. 1*A*).

**Fig. 1.**
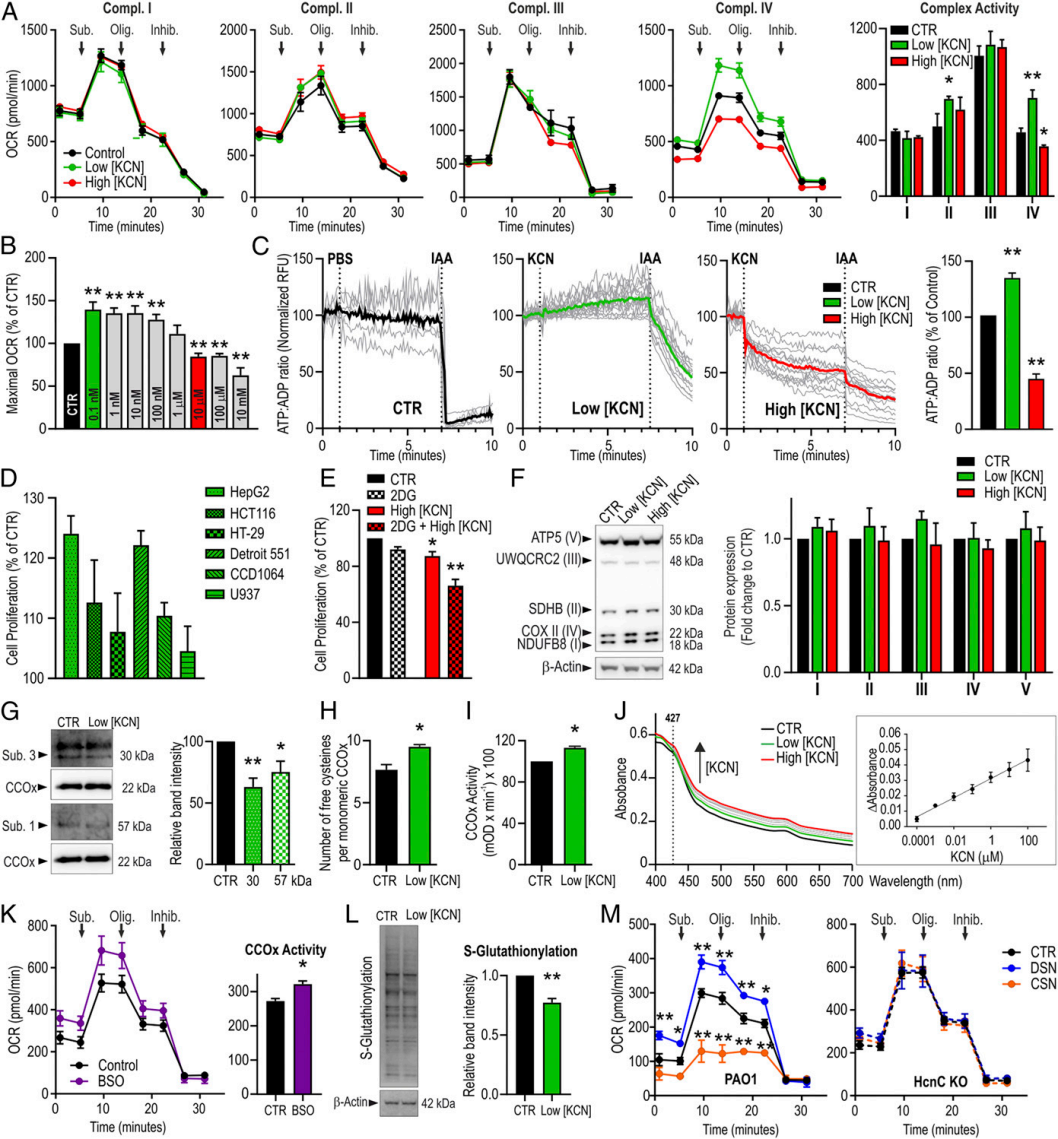
(*A–C*) Low cyanide concentrations stimulate, while high cyanide concentrations suppress mitochondrial electron transport, CCOx activity, and ATP biosynthesis in HepG2 cells, as measured by extracellular flux analysis (*A* and *B*) or a fluorescent ATP:ADP biosensor (*C*). KCN in *A* and *C* was applied at 0.1 nM (low concentration) or 10 µM (high concentration). In *C*, after KCN (0.1 nM or 10 µM) the GAPDH inhibitor iodoacetamide (IAA; 1 mM) was applied, which produced a rapid decrease in ATP levels. In *B*, a broad range of cyanide concentrations was tested (0.1 nM to 10 mM). (*D*) Cyanide (0.1 nM, 6 h) increases proliferation in various human cell lines. (*E*) High cyanide concentrations (10 µM, 6 h), especially after pretreatment of the cells with the glycolysis inhibitor 2-deoxyglucose (2DG; 5 mM, 2h), suppress HepG2 cell proliferation. (*F*) Cyanide exposure (0.1 nM or 10 µM, 6 h) does not affect mitochondrial complex expression levels in HepG2 cells. (*G*) Cyanide (0.1 nM) catalyzes constitutive *S*-glutathionylation removal from purified CCOx, evidenced by western blotting (30- and 57-kDa subunits; *G*) and the DTNB assay (*H*); these effects are associated with an increase in CCOx-specific activity (*I*). (*J*) Cyanide (0.1 nM to 100 µM) induces spectral changes in CCOx. (*K* and *L*) Pharmacological suppression of cellular glutathione levels with buthionine sulfoximine (BSO; 0.5 mM, 24 h) increases CCOx activity in HepG2 cells and reduces protein glutathionylation in whole-cell lysates. (*M*) HepG2 cells exposed to diluted supernatants (DSNs) of *P. aeruginosa* (PAO1; cyanide: ∼20 nM) stimulate CCOx activity, while concentrated supernatants (CSNs; cyanide: ∼2 µM) inhibit CCOx; these effects were absent when supernatants of an HcnC-KO *Pseudomonas* (a mutant strain, deficient in cyanide production) were used (HcnC KO). Control: CTR; oxygen consumption rate: OCR; phosphate-buffered saline: PBS; glyceraldehyde 3-phosphate dehydrogenase: GAPDH; relative fluorescence unit: RFU; UWQCRC2: cytochrome B-C1 complex subunit 2, (mitochondrial); SDHB: succinate dehydrogenase complex iron sulfur subunit B; COX II: cytochrome C oxidase subunit II; NDUFB8: NADH:Ubiquinone oxidoreductase subunit B8; DTNB: 5,5'-dithiobis-(2-nitrobenzoic acid). *P < 0.05; **P < 0.01.

Low concentrations of cyanide stimulated proliferation in HepG2 cells and in a panel of additional human cell lines (Fig. 1*D*). High concentrations of cyanide resulted in the expected inhibition of cell proliferation; this effect was more pronounced after glycolysis was pharmacologically blocked with 2-deoxyglucose (Fig. 1*E*), consistent with the fact that in cultured cells, both glycolysis and mitochondrial activity contribute to ATP generation (1, 6). Cyanide did not affect the expression of Complex IV or other mitochondrial complexes (Fig. 1*F*).

The binding of low concentrations of cyanide reduced the basal glutathionylation of CCOx—chiefly, on its 30- and 57-kDa catalytic subunits (i.e., Subunits III and I) (Fig. 1*G*). The removal of this constitutive glutathionylation is reflected in an increase in free cysteines (Fig. 1*H*) and in higher enzymatic activity (Fig. 1*I*). Glutathionylation is typically an inhibitory posttranslational protein modification (7); in CCOx, the solvent-accessible Cys498 (Subunit I) and Cys115 (Subunit III) are the likely constitutive glutathionylation sites (8). We propose that low concentrations of cyanide stimulate CCOx by removing its constitutive, inhibitory glutathionylation. Cyanide is readily reactive toward protein disulfides (9); according to our proposed model, it catalyzes CCOx deglutathionylation by interacting with its –SSG disulfide, thus releasing free glutathione.

As expected, high (inhibitory) concentrations of cyanide produced the previously reported spectral change of CCOx (Fig. 1*J*), but also, the low (activating) concentrations of cyanide produced detectable spectral changes (Fig. 1*J*). We interpret the latter finding as an indication that the removal of Complex IV’s basal glutathionylation induces a conformational change, affecting its spectral character.

Depletion of endogenous glutathione enhanced cellular bioenergetics (Fig. 1*K*), indirectly supporting the concept that endogenous glutathionylation of CCOx (and possibly, of additional enzymatic targets) tonically suppresses cellular energy generation. The decrease in protein glutathionylation after glutathione depletion was also confirmed in whole-cell lysates (Fig. 1*L*).

*Pseudomonas aeruginosa* is a typical cyanide-producing bacterium; cyanide produced by this bacterium has previously been implicated in the context of bacterial quorum sensing (10) as well as in mediating bacterial–host interactions in a cytotoxic context (11–13). Transfer of diluted bacterial supernatants to HepG2 cells stimulated cellular bioenergetics, while concentrated supernatants produced the expected inhibitory effects. These effects were absent with supernatants from a mutant strain lacking its cyanide-producing enzyme (Fig. 1*M*).

There are several confirmed or putative mammalian enzymes involved in cyanide synthesis and/or metabolism (2–5). In HepG2 cells, we have confirmed the expression of six enzymes: epoxide hydrolase, β-glucosidase, myeloperoxidase, methylmalonic aciduria and homocystinuria type C protein, 3-mercaptopyruvate sulfurtransferase, and thiosulfate sulfurtransferase (Fig. 2). Interestingly, exposure of the cells to cyanide produced a biphasic regulation of myeloperoxidase: up-regulation at low and down-regulation at high cyanide concentrations (Fig. 2).

**Fig. 2.**
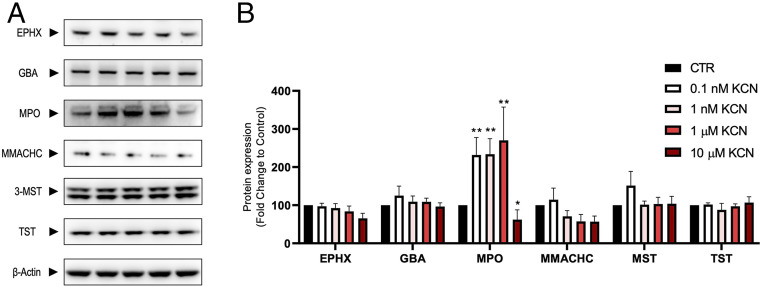
Expression of epoxide hydrolase (EPHX), β-glucosidase (GBA), myeloperoxidase (MPO), methylmalonic aciduria and homocystinuria type C protein (MMACHC), 3-mercaptopyruvate sulfurtransferase (3-MST), and thiosulfate sulfurtransferase (TST) in HepG2 cells under baseline conditions and after exposure to various concentrations of cyanide for 6 h. β-actin was used as loading control. (*A*) Representative western blots; (*B*) densitometry analysis. Note the biphasic changes in MPO expression in response to cyanide. CTR: control conditions, in the absence of added cyanide. *P < 0.05; **P < 0.01.

Thus, cyanide, at low concentrations, acts as a mitochondrial activator and stimulatory bioenergetic factor. Its effects are associated with the stimulation of CCOx activity and an elevation of intracellular ATP, leading to a stimulation of cell proliferation. The bell-shaped bioenergetic effect is also noted with supernatants of a cyanide-producing bacterium, raising the possibility that cyanide may serve various concentration-dependent signaling roles in bacteria–host interactions.

Mammalian cells synthesize various small gaseous diffusible molecules, which, in turn, serve various regulatory functions. From these molecules, nitric oxide (NO), carbon monoxide (CO), and hydrogen sulfide (H_2_S) are typically viewed as the three principal gasotransmitters (14). Cyanide may be the “fourth gasotransmitter” since 1) it is in the gaseous state in physiological fluids; 2) it is produced by mammalian enzymes; 3) it is detectable in low-micromolar concentrations in various cells (2–5); 4) its levels are dynamically regulated; and 5) at low concentrations, it can exert regulatory biological actions.

The current findings open up several follow-up directions related to the regulation and action of endogenously produced cyanide in mammalian cells in health and disease.

## Materials and Methods

Cell culture, cell proliferation, western blotting, intracellular ATP:ADP fluorescent biosensor assays, measurement of purified bovine heart CCOx activity, and experiments with *P. aeruginosa* supernatant transfers are presented in *SI Appendix*.

## Supplementary Material

Supplementary File

## Data Availability

All study data are included in the article and/or *SI Appendix*.
