# Supplementary material for: Physiological concentrations of cyanide stimulate mitochondrial Complex IV and enhance cellular bioenergetics
Source: Proc Natl Acad Sci U S A. 2021 May 10;118(20):e2026245118. doi: 10.1073/pnas.2026245118 (PMC8157914; doi:10.1073/pnas.2026245118)
Supplement: Supplementary File [file pnas.2026245118.sapp.pdf]

SI Appendix: Extended Methods

Cell culture.

HCT116 and HT-29 were cultured in McCoy's 5A modified medium (Gibco, Thermo Fisher Scientific, Basel, Switzerland). U937 cells were differentiated for 48 h using PMA (100 ng/ml) in RPMI medium (Gibco). HepG2, Detroit 551 and CCD1064 cells were grown in DMEM (Gibco). Culture media were supplemented with 10% heat-inactivated fetal bovine serum (FBS), 100 IU/ ml of penicillin and 100 µg/ml of streptomycin. Cells were maintained at 37 °C in a humidified incubator with 5% CO_2 and 95% air. Cells were subcultured when 80 – 90% confluent and seeded at a ratio of 1:4.

Pharmacological treatments.

Potassium cyanide (KCN, Sigma-Aldrich, St Louis, MO, USA) was added to the cells at various concentrations and measurements were performed 1h later (Complex I,II,III or IV activity measurements and the Extracellular Flux Analysis or 6 h later (cell proliferation assays and expression analysis of mitochondrial complexes and of cyanide-regulating enzymes).

To inhibit glycolysis, cells were pretreated for 2 h with 5 mM 2-deoxyglucose (2DG, Sigma- Aldrich) followed by KCN administration (10 µM, 6 h).

Cells were treated with 0.5 mM of buthionine sulfoximide (BSO, 24h) to inhibit glutathione biosynthesis.

Supernatant from wild-type PAO1 and HcnC KO bacteria was sterile-filtered and diluted 1:1 to 1:160 with DMEM medium. Cells were incubated with increasing concentration of bacterial supernatant for 6 h and then subjected to Extracellular Flux analysis.

The concentration of cyanide in the bacterial supernatants was quantified using a cyanide detection kit (Macherey-Nagel, Düren, Germany). Bacterial supernatants from PAO1 contained ~4 µM cyanide; in HcnC KO bacterial supernatants, cyanide concentrations were below the limit of detection of the assay.

Cellular bioenergetic measurements using Extracellular Flux Analysis.

Cellular bioenergetics was measured by the Extracellular Flux Analysis method (1,2). Cells were seeded on cell culture microplates at 20,000/well. For analysis of mitochondrial respiration ("Mitostress Assay"), the cells were washed twice with DMEM medium (pH 7.4), supplemented with L-glutamine (2 mM, Gibco), sodium pyruvate (1 mM, Sigma) and glucose (10 mM, Sigma). After 1h incubation at 37°C in CO_2-free incubator, cellular oxygen consumption rate (OCR) after oligomycin (1 µM) was used to assess ATP production rate and OCR after carbonyl cyanide-4-trifluoromethoxy phenylhydrazone (FCCP, 0.8 µM) was used to assess maximal mitochondrial respiratory capacity. Antimycin A (0.5 µM) and rotenone (0.5 µM) were used to inhibit the flux of electrons through Complex III and I. Residual non-mitochondrial OCR, is considered to be due to cytosolic oxidase enzymes.

The specific activity of the different mitochondrial complexes in permeabilized cells was measured by the Extracellular Flux Analysis method as described (2). Briefly, cells were washed twice with mannitol-sucrose-BSA (MAS-BSA) buffer (70 mM sucrose, 220 mM mannitol, 10 mM KH_2PO_4, 5 mM $MgCl_2$, 2 mM HEPES, 1 mM EGTA and 4 mg/ml fatty acid-free BSA; pH 7.2). OCR was measured twice at steady state and following each port injection, which were prepared as follows: (i) Complex I: A) saponin (50 µg/ml, Sigma)/ADP (1 mM, Sigma)/FCCP (1 µM, Sigma)/Pyruvate-Malate (5-2.5 mM, Sigma); B) oligomycin (1 µg/ml, Sigma); C) rotenone (1 µM, Sigma); (ii) Complex II: A) saponin (50 µg/ml)/ADP (1 mM)/FCCP (1 µM)/succinate (10 mM, Sigma)/rotenone (1 µM); B) oligomycin (1 µg/ml); C) rotenone-AMA (1-20 µM, Sigma); (iii) Complex III: A) saponin (50 µg/ml)/ADP (1 mM)/FCCP (1 µM)/duroquinol (0.5 mM, TCI America); B) oligomycin (1 µg/ml); C) AMA (20 µM); (iv) Complex IV: A) saponin (50 µg/ml)/tetramethyl-p-phenylene diamine (0.5 mM,

Sigma) / ascorbate (2 mM)/FCCP (1 μM)/ADP (1 mM); B) oligomycin (1 μg/ml); C) sodium azide (20 mM, Sigma).

Ratiometric intracellular ATP:ADP measurements.

To measure changes in cellular energy state, we used PercevalHR, a ratiometric intracellular ATP:ADP fluorescent biosensor (3). HepG2 cells were seeded on collagen coated glass bottom dishes (Mattek Corporation) and transfected with a Perceval High Resolution (PercevalHR) reporter plasmid using TransIT®-LT1 reagents (Mirus Bio). GW1-PercevalHR was a gift from Gary Yellen (Addgene plasmid # 49082). A DMI6000 inverted confocal microscope integrated to a Leica TCS-SP5 workstation was used to examine fluorescence signals. The following excitation wavelengths were used to illuminate the fluorophore: 405 nm for and 488 nm. Emissions were recorded with a 20x objective and bandpass filters of 509–578 nm. Fluorescence images were collected every 5 s. The drugs (KCN, 0.1 nM or 10 μM final concentration, iodoacetamide; IAA, 1 mM final concentration) were added to the DPBS solution (Gibco # 14040117). Regions of interest (ROIs) were placed within 1–10 μm along the free edge of the cells. Fluorescence values were calculated after background subtraction (fluorescence intensity of regions without cells). By taking the ratio of the fluorescence at two different excitation wave lengths (F_{high}/F_{low}), the ratiometric signal reports the occupancy of PercevalHR independent of the amount of sensor protein. Fluorescence intensity ratios were normalized in each experiment to the averaged basal value preceding the treatment period. Individual trajectories and averages were plotted.

Cell proliferation assay.

The cell proliferation ELISA BrdU (colorimetric) assay was performed using Corning Costar TC-Treated flat-bottomed 96-well plates (4). Following pharmacological treatments, cells were incubated with 10 μM BrdU labelling solution for 4 h at 37 °C in a humidified incubator with 5% CO_2 and 95% air. The assay is based on the principle that pyridine analogue BrdU incorporates (in place of thymidine) into the newly synthesized DNA strands of proliferating cells. BrdU incorporation was detected by immune-peroxidase staining and a subsequent colorimetric substrate reaction as per the manufacturer's protocol. Plates were read at 450 and 690 nm (reference wavelength) using an Infinite 200 PRO microplate reader. Developed color and absorbance values reflect the amount of DNA synthesis, which correlates with the number of proliferating cells.

Western blotting.

Cells were washed once with ice-cold 1X PBS and harvested in pre-cooled 1X ELISA lysis buffer previously supplemented with protease/phosphatase inhibitor cocktail (1X). Following 2 freeze/thaw cycles, whole-cell lysate was collected, sonicated for 5 min (30 sec ON / 30 sec OFF) in an ultrasonic water-bath, and the total protein was extracted by centrifugation at 16,000 x *g* at 4 °C for 15 min. The Pierce Coomassie Plus Bradford protein assay was used to quantify the protein concentration of the samples.

Expression levels of various mitochondrial electron transport chain complexes from cell homogenates were quantified with the total OXPHOS human antibody cocktail containing 5 monoclonal antibodies, one each against Complex I subunit NDUFB8 (ab110242), Complex II subunit 30kDa (ab14714), Complex III subunit Core 2 (ab14745), Complex IV subunit II (ab110258), and ATP synthase subunit alpha (ab14748) (1).

For the determination of S-glutathionylation from purified Complex IV or cell homogenates, protein samples were added to Laemmli buffer (in non-reducing conditions) supplemented with 25 mM N-ethyl maleimide (NEM) and incubated for 5 min at 95°C. Proteins were transferred in a PVDF membrane and blocked with milk (5% w/v) supplemented with 25 mM NEM. S-glutathionylation was detected with anti-SSG primary antibody (Virogen; 1:500)

then reacted with horseradish-peroxidase-conjugated secondary antibodies (5). Complex IV and cell homogenate signals were normalized with COX-II (Abcam, 1:1000) and actin (Cell Signalling, 1:2000) antibody, respectively.

For the detection of various mammalian cyanide-producing and/or cyanide-metabolizing enzymes (6-14) under basal conditions or after exposure of HepG2 cells to various concentrations of cyanide for 6 hours, Western blotting was performed using the following antibodies: anti-epoxide hydrolase antibody (rabbit polyclonal; ab96774 – 1:1000 in MILK 5% Tween-TBS – molecular weight: 53 kDa); anti-myeloperoxidase antibody (rabbit monoclonal; Abcam, ab109116 - 1:1,000 in milk 5% Tween-TBS – molecular weight: 84 kDa); anti-β-glucosidase (GBA) antibody (mouse polyclonal; ab88300 – 1:1,000 in milk 5% Tween-TBS – molecular weight: 60 kDa); anti-methylmalonic aciduria and homocystinuria type C protein (MMACHC/CblC) antibody (rabbit polyclonal; Abcam, ab96195 – 1:1,000 in milk 5% Tween-TBS – molecular weight: 32 kDa); anti-3-mercaptopyruvate sulfurtransferase (3-MST) antibody (rabbit polyclonal; Abcam, ab224043 – 1:1,000 in milk 5% Tween-TBS – molecular weight: 33 kDa); anti-thiosulfate sulfurtransferase (TST) antibody (rabbit polyclonal; Abcam, ab231248 – 1:500 in milk 5% Tween-TBS –molecular weight: 33 kDa) and loading control: anti-β-actin antibody (mouse monoclonal; Sigma-Aldrich, A1978 – 1:3,000 in milk 5% Tween-TBS – molecular weight: 42 kDa).

Cyanide binding to Complex IV (Cytochrome C Oxidase, CCOx).

Complex IV titration, adapted from Jones and colleagues (17) was monitored at room temperature by UV-visible absorption spectroscopy, in an Infinite M200 Pro spectrophotometer (Tecan, Männedof, Switzerland) equipped with magnetic stirrer, using a rubber-cap sealed quartz cuvette. Purified Complex IV from bovine heart (Sigma, 5 µM) in 100 mM potassium phosphate buffer, pH 7.4 supplemented with 0.5 % (w/v) N-dodecyl β maltoside (Buffer A) was reduced with sodium dithionite (15 mM final concentration) and washed through a MiniTrap G-25 column (pre-equilibrated with buffer A). After each KCN addition with gas-tight Hamilton syringes, the solution was stirred for 10 min, and cyanide binding to heme a3 was monitored at 427 nm.

Quantification of free thiols of purified bovine Complex IV.

Free protein thiols were quantitated with the DTNB method (4). Purified Complex IV from bovine heart (Sigma, 1 µM, in Buffer A) was reduced with TCEP (0.5 mM final concentration) and on ice for 1 hour. The excess of TCEP was washed out with a MiniTrap G-25 column, followed by incubation for 1h (room temperature) with 0.1 nM KCN or vehicle. The samples were further washed with a MiniTrap G-25 column and eluted in 500 µl of buffer A. 450 µl of the eluate were transferred in an Eppendorf tube and supplemented with 50 µl of 10 mM DTNB (1 mM final concentration) and incubated for 30 min at RT in the absence of light. The remaining 50 µl was used for the activity assay (see below). Spectra were obtained in an Infinite M200 Pro spectrophotometer (Tecan, Männedof, Switzerland) with a quartz cuvette. 2-nitro-5-thiobenzoic acid anion formation was estimated by analyzing absorbance values at 412 nm ($\varepsilon_{412\ nm} = 14{,}150\ M^{-1}\ cm^{-1}$).

Determination of the specific enzymatic activity of Complex IV.

The activity of purified Complex IV was measured using the colorimetric ELISA Complex IV enzyme activity microplate assay kit (Abcam). Each well of the ELISA plate contained 0.2 µg of protein in 200 µl of assay buffer (provided by the manufacturer) and was incubated with various concentrations of KCN or vehicle at room temperature for 3 hours in the absence of light. Complex IV activity was determined colorimetrically by monitoring the oxidation of reduced cytochrome c by the absorbance change at 550 nm over 2 hours at 30 °C.

1. T. Panagaki, E. B. Randi, F. Augsburger, C. Szabo, Overproduction of H_2S, generated by CBS, inhibits mitochondrial Complex IV and suppresses oxidative phosphorylation in Down syndrome. *Proc. Natl. Acad. Sci. USA*. **116**, 18769-18771 (2019).
2. J. K. Salabei, A. A. Gibb, B. G. Hill, Comprehensive measurement of respiratory activity in permeabilized cells using extracellular flux analysis. *Nat. Protoc.* **9,** 421-438 (2014).
3. J. Berg, Y. P. Hung, G. Yellen, A genetically encoded fluorescent reporter of ATP: ADP ratio. *Nat. Methods* **6**, 161-166 (2009).
4. K. Zuhra *et al*., Mechanism of cystathionine-β-synthase inhibition by disulfiram: The role of bis(N,N-diethyldithiocarbamate)-copper(II). *Biochem. Pharmacol.* **182**, 114267 (2020).
5. B. G. Hill, K. V. Ramana, J. Cai, A. Bhatnagar, S. K. Srivastava, Measurement and identification of S-glutathiolated proteins. *Methods Enzymol*. **473**, 179-197 (2010).
6. B. Vennesland *et al*., Cyanide metabolism. *Fed. Proc*. **41**, 2639-2648 (1982).
7. A. R. Dahl, The cyanide-metabolizing enzyme rhodanese in rat nasal respiratory and olfactory mucosa. *Toxicol. Lett*. **45**, 199-205 (1989).
8. D. W. Porter, S. I. Baskin, Specificity studies of 3-mercaptopyruvate sulfurtransferase. *J. Biochem. Toxicol.* **10**, 287-292 (1995).
9. N. Nagahara, T. Ito, M. Minami, Mercaptopyruvate sulfurtransferase as a defense against cyanide toxication: molecular properties and mode of detoxification. *Histol. Histopathol*. **14**, 1277-1286 (1999).
10. P. G. Gunasekar, J. L. Borowitz, J. J. Turek, D. A. Van Horn, G. E. Isom, Endogenous generation of cyanide in neuronal tissue: involvement of a peroxidase system. *J. Neurosci. Res.* **61**, 570-575 (2000).
11. H. Wang, B. Chanas, B. I. Ghanayem, Cytochrome P450 2E1 (CYP2E1) is essential for acrylonitrile metabolism to cyanide: comparative studies using CYP2E1-null and wild-type mice. *Drug Metab. Dispos*. **30**, 911-917 (2002).
12. K. Michelin *et al*., Biochemical study on beta-glucosidase in individuals with Gaucher's disease and normal subjects. *Clin. Chim. Acta* **343**, 145-153 (2004).
13. R. Cipollone, P. Visca, Is there evidence that cyanide can act as a neuromodulator? *IUBMB Life* **59**, 187-189 (2007).
14. J. Kim, C. Gherasim, R. Banerjee, Decyanation of vitamin B12 by a trafficking chaperone. *Proc. Natl. Acad. Sci. USA*. **105**, 14551-14554 (2008).
15. Y. Zhang, K. Zhu, X. Miao, X. Hu, T. Wang, Identification of beta-glucosidase 1 as a biomarker and its high expression in hepatocellular carcinoma is associated with resistance to chemotherapy drugs. *Biomarkers* **21**, 249-256 (2016).
16. C. Delporte *et al*., Myeloperoxidase-catalyzed oxidation of cyanide to cyanate: A potential carbamylation route involved in the formation of atherosclerotic plaques? *J. Biol. Chem.* **293**, 6374-6386 (2018).
17. M. G. Jones *et al*., A re-examination of the reactions of cyanide with cytochrome c oxidase. *Biochem. J.* **220**, 57-66 (1984).
